# Palmitoylation of acetylated tubulin and association with ceramide-rich platforms is critical for ciliogenesis

**DOI:** 10.1194/jlr.RA120001190

**Published:** 2021-01-07

**Authors:** Priyanka Tripathi, Zhihui Zhu, Haiyan Qin, Ahmed Elsherbini, Simone M. Crivelli, Emily Roush, Guanghu Wang, Stefka D. Spassieva, Erhard Bieberich

**Affiliations:** 1 Department of Physiology, University of Kentucky College of Medicine, Lexington, KY, USA; 2 Veterans Affairs Medical Center, Lexington, KY, USA

**Keywords:** acetylation, Arl13b, lipids, cell biology, ceramide, palmitoylation, cilia, tubulin, microtubules, lipid rafts, 2BPA, 2-bromo palmitic acid, CAP, ceramide-associated protein, CRP, ceramide-rich platform, FB1, fumonisin B1, iPS, induced pluripotent stem, NP, neuroprogenitor, PAz, palmitic azide, PLA, proximity ligation assay, TAP, Tris-acetate-phosphate

## Abstract

Microtubules are polymers composed of αβ-tubulin subunits that provide structure to cells and play a crucial role in in the development and function of neuronal processes and cilia, microtubule-driven extensions of the plasma membrane that have sensory (primary cilia) or motor (motile cilia) functions. To stabilize microtubules in neuronal processes and cilia, α tubulin is modified by the posttranslational addition of an acetyl group, or acetylation. We discovered that acetylated tubulin in microtubules interacts with the membrane sphingolipid, ceramide. However, the molecular mechanism and function of this interaction are not understood. Here, we show that in human induced pluripotent stem cell–derived neurons, ceramide stabilizes microtubules, which indicates a similar function in cilia. Using proximity ligation assays, we detected complex formation of ceramide with acetylated tubulin in *Chlamydomonas reinhardtii* flagella and cilia of human embryonic kidney (HEK293T) cells, primary cultured mouse astrocytes, and ependymal cells. Using incorporation of palmitic azide and click chemistry–mediated addition of fluorophores, we show that a portion of acetylated tubulin is S-palmitoylated. S-palmitoylated acetylated tubulin is colocalized with ceramide-rich platforms in the ciliary membrane, and it is coimmunoprecipitated with Arl13b, a GTPase that mediates transport of proteins into cilia. Inhibition of S-palmitoylation with 2-bromo palmitic acid or inhibition of ceramide biosynthesis with fumonisin B1 reduces formation of the Arl13b-acetylated tubulin complex and its transport into cilia, concurrent with impairment of ciliogenesis. Together, these data show, for the first time, that ceramide-rich platforms mediate membrane anchoring and interaction of S-palmitoylated proteins that are critical for cilium formation, stabilization, and function.

Ceramide is a sphingolipid that was shown to form membrane microdomains also known as “ceramide rafts” or “ceramide-rich platforms (CRPs)” (1, 2). CRPs are proposed to function as platforms for signaling proteins such as receptors and kinases. However, it is matter of an ongoing debate how CRPs interact with the proteins that are thought to be enriched in them. In one model, ceramide interacts directly with a specific binding site in the ceramide-associated protein (CAP) (2, 3). If the CAP is a cytosolic protein that binds to CRPs, the binding site either “digs” into the membrane, which was proposed for atypical PKCζ/λ by a mechanism similar to diacylglycerol binding of classical PKCα (4, 5, 6), or the CAP somehow attaches to the membrane surface without leaflet intrusion. In this case, the attachment of the CAP to the CRP may not be mediated by ceramide itself, but ceramide gets “lifted” into a binding site of the protein. This was shown for CERT, a protein that binds to a palmitoylated ER protein and phosphatidylinositol phosphate at the Golgi to transport ceramide from the ER to the Golgi apparatus (7). Indirect binding and engulfment of ceramide into its binding site may also be the mechanism by which ceramide interacts with other proteins such as the inhibitor protein of phosphatase 2a, StarD7, p53, and enzymes that use ceramide as the substrate (8, 9, 10).

Recently, we proposed a third mechanism for ceramide binding to a cytosolic protein. We found that translocation of the peroxisomal protein Hsd17b4 is regulated by binding to ceramide in mitochondrial-associated membranes (11). Molecular modeling predicted that the Scp-2–like domain at the C-terminus of Hsd17b4 interacts with the sphingosine residue of ceramide, while the fatty acid acyl chain resides in the membrane. We noted that this “splayed-chain” binding of ceramide resembles anchoring of cytosolic proteins to cellular membranes by covalently attaching fatty acyl residues such as myristoyl or palmitoyl residues. Our observation prompted us to investigate if palmitoylation of proteins is another mechanism to anchor proteins to CRPs. Most recently, a study on the lipid raft proteins in Niemann-Pick disease showed that plasma membrane association of more than 100 palmitoylated proteins is critically relying on the presence of ceramide (12). Therefore, it is reasonable to assume that CRPs mediate membrane association of palmitoylated proteins.

We tested this assumption with two proteins, acetylated tubulin and Arl13b in cilia. Previously, we showed that that the ciliary membrane of *Chlamydomonas reinhardtii* and mammalian cells is highly enriched with ceramide (phytoceramide in *C. reinhardtii*) (1, 13, 14, 15, 16). In addition, we showed that ceramide depletion destabilizes microtubules in cilia and neuronal processes, while ceramide supplementation rescued ciliogenesis and promoted neuronal process growth (14). Because it was shown that tubulin can be palmitoylated at a cysteine residue (S-palmitoylation) in the C-terminus (17), we hypothesized that S-palmitoylation of tubulin is a mechanism to interact with ceramide and that this interaction is critical for ciliogenesis. Cilium growth relies on transport of acetylated tubulin to the cilium tip and therefore, binding to Arl13b, a small GTPase with ciliary transport function that depends on Arl13b being S-palmitoylated (18, 19). We tested if S-palmitoylation of both acetylated tubulin and Arl13b is required for complex formation and anchoring of the protein complex to ceramide in the ciliary membrane.

Most recently, we introduced novel labeling techniques to detect and visualize ceramide binding to CAPs in CRPs and developed them further to determine if S-palmitoylation mediates binding to ceramide (1). The basis of these techniques is the proximity ligation assay (PLA), which allows for visualization of protein complexes with ceramide if they are closer than 40 nm to each other (1, 20). We combined this assay with metabolic labeling of S-palmitoylated proteins using palmitic azide (PAz), the incorporation of which into these proteins is mediated by S-palmitoyl transferase (21). Specificity of this reaction and significance of S-palmitoylation were tested with 2-bromo palmitic acid (2BPA), a covalent inhibitor of S-palmitoyl transferase (22). S-palmitoylated proteins are detected by click chemistry–mediated addition of a fluorophore and the PLA using antibodies against the protein and the fluorophore. Using this technique, a recent study found that a portion of acetylated tubulin is S-palmitoylated (23). By complementing this technique using the PLA with anti-ceramide antibody and cross-linking of the S-palmitoylated protein to a photoactivatable ceramide analog, we show here that acetylated tubulin and Ar113b form complexes that associate with CRPs in cilia. Protein complex formation is confirmed by coimmunoprecipitation assays and the function of S-palmitoylation and ceramide is supported by loss-of-function assays using 2BPA and fumonisin B1 (FB1), an inhibitor for ceramide generation, respectively. Our data show that S-palmitoylation and ceramide are both required for complex formation between acetylated tubulin and Arl13b, which is critical for cilium function.

## Materials and methods

### Materials

DMEM was from Hyclone (Logan, UT) and FBS was from Atlanta Biologicals (Flowery Branch, GA). Penicillin and streptomycin were (Cat#15146) purchased from Gibco (Grand Island, NY). Protease and phosphatase inhibitors (cat# A32961) were from Thermo Fisher Scientific (West Columbia, SC). N-(9-(3-pent-4-ynyl-3-H-diazirin-3-yl)-non-anoyl)-D-erythro-shpingosine (pacFACer, cat# 900404) was obtained from Avanti Polar Lipids (Alabaster, AL). FB1 was from Enzo Life Sciences (Farmingdale, NY). Anti-acetylated tubulin rabbit IgG (cat# 5335S, 1:100 for PLAs) was from Cell Signaling Technology. 2-Bromo hexadecanoic acid (2BPA, cat# 238422), anti-acetylated tubulin mouse IgG (cat# T6793, 1:3,000 for immunocytochemistry and PLA; 1:5,000 for immunoblot), and SDS-PAGE sample buffer (cat# S3401) were from Sigma-Aldrich (St. Louis, MO). Anti-ceramide rabbit IgG (1:100 for immunocytochemistry and PLA) was generated in our laboratory (27). Anti-ceramide mouse IgM was from Glycobiotech (1:100, MAS0014, Kuekels, Germany). Anti-Arl13b rabbit IgG (cat# 17711-1-AP, 1:100 for PLAs) was from ProteinTech. Anti-Cy5 mouse IgG (cat# sc-166896, 1:100 for PLA), anti-Arl13b mouse IgG (sc-515784, 1:100 for PLA), and Protein A-Agarose beads (sc-2001) were from Santa Cruz Biotechnology (Dallas, TX). Azido palmitic acid (Catalog#1346) and Cy5 DBCO was from Click Chemistry Tools (Scottsdale, AZ). Duolink PLA probe anti-rabbit plus (DUO92002), Duolink PLA probe anti-mouse minus (DUO92004), Duolink detection reagent red (DUO92008), and Duolink detection reagent green (DUO92014) were purchased from Thermo Fisher Scientific (West Columbia, SC).

### Cell cultures

*C. reinhardtii* (wild-type strain CC-125 obtained from the Chlamydomonas Resource Center at the University of Minnesota, St. Paul, MN) was grown in the Tris-acetate-phosphate (TAP) growth medium (Gibco) in six-well plates or 250 ml Erlenmeyer flasks with a light/dark cycle of 14:10 h at room temperature. All experiments using mice to generate primary cell cultures were carried out according to an Animal Use Protocol approved by the Institutional Animal Care and Use Committee at the University of Kentucky. The primary cell culture of mixed glial cells was isolated from brains of 1-day-old C57BL/6 wild-type mouse pups following a protocol previously published (24). In brief, brains were dissociated in PBS containing 0.1 M glucose, passed through a 40 μm filter, and plated in T-25 flasks in DMEM supplemented with 10% FBS and 1% penicillin/streptomycin solution at 37°C in a humidified atmosphere containing 5% CO_2_. After 7 days in culture, mixed glial cells were transferred to poly-D-lysine–coated cover slips in 24-well plates, and astrocytes and ependymal cells were enriched following protocols as previously published (16, 25). To enrich astrocytes, adherent cells were cultured in DMEM as described above. To enrich ependymal cells, the medium was changed to MEMα (Life Technologies) supplemented with BSA (0.5 mg/ml), insulin (10 μg/ml; bovine), transferrin (5.5 μg/ml), sodium selenite (6.7 ng/ml), and human thrombin (1 U/ml) (16). Cultures of >70% proportion of ependymal cells were visible after another 7 d of cultivation in the thrombin-supplemented medium (purity was checked by immunocytochemistry for multiple versus primary cilia and glial markers such as glial fibrillary acid protein). HEK293T cells (received from Dr Lin Mei, Augusta University, Augusta, GA) were maintained in DMEM with 10% FBS and 1% penicillin/streptomycin at 37°C in a humidified atmosphere containing 5% CO_2_. To cultivate human induced pluripotent stem (iPS) cell–derived neuroprogenitor (NP) cells, the ReNcell VM Human NP cell line was obtained from Millipore (Temecula, CA, Cat# SCC008). Cells were maintained according to the supplier's and previously published protocols (14, 26). Briefly, cells were expanded on laminin-coated 100 mm tissue culture dishes (Corning) in ReNcell NSC maintenance medium (Millipore) supplemented with 20 ng/mL fibroblast growth factor-2 and 20 ng/mL epidermal growth factor (Millipore). The medium was changed daily during the maintenance period. The cells were passaged once a week using Accutase (Millipore). Cells were then differentiated by seeding them at around 60% confluency on freshly laminin-coated dishes and growing overnight in the presence of growth factors, followed by withdrawal of growth factors. The media were replaced every other day up to 10 days during the differentiation period.

### Metabolic labeling of S-palmitoylated proteins and treatment with 2BPA and FB1

*C. reinhardtii* and mammalian cells were grown in suspension and adherent on glass cover slips, respectively, as described in the previous section. They were treated with 50 μM PAz (1:1,000 from a 50 mM stock in DMSO) in the TAP growth medium (*C. reinhardtii*) or serum-free DMEM containing 1% fatty acid-free BSA (mammalian cells) and incubated overnight. Cells were then processed for click chemistry–mediated addition of fluorophores and protein chemistry or immunocytochemistry analyses as described in the following sections. To prevent S-palmitoylation, *C. reinhardtii* were preincubated for 4 h with 100 μM 2BPA (1:1,000 from a 100 mM stock in DMSO) by dilution in a fresh medium (TAP or serum-free DMEM containing 1% fatty acid-free BSA). The pH of the medium was adjusted to neutral before its addition to cells. 2BPA was present during incubation with PAz. To prevent S-palmitoylation in primary cultured astrocytes and ependymal cells, the concentration of 2BPA was lowered to 10 μM. Cells were viable and did not show signs of toxicity at these concentrations. To prevent ceramide biosynthesis, mammalian cells were preincubated for 48 h with 10 μM FB1 (1:1,000 from a 10 mM stock in water). Then medium was exchanged to fresh serum-free medium containing 1% fatty acid-free BSA, 10 μM FB1, and 50 μM PAz and cells further incubated overnight for further processing. Addition of C24:1 ceramide at a concentration of 2 μM in the medium was achieved by 1:1,000 dilution from a 2 mM solution in ethanol supplemented with 2% dodecane (final concentration) as previously described (14). Controls were treated with 1:1000-diluted vehicle without ceramide.

### Immunoprecipitation and coimmunoprecipitation of S-palmitoylated proteins

*C. reinhardtii* and HEK293T cells were treated with 50 μM PAz as described in the previous section. Untreated control and treated human embryonic kidney cells were washed with ice-cold PBS and harvested by centrifugation at 2,000 *g* for 5 min at 4°C. *C. reinhardtii* cells were centrifuged at 1,000 *g* for 10 min at RT to recover the pellet. Cells were lysed in 1 ml of lipid binding buffer (20 mM Tris-HCl, 150 mM NaCl, 1 mM EDTA, pH 7.5) supplemented with protease/phosphatase inhibitors at 1:100 (Roche) with 1% Triton X-100, sonicated, and centrifuged at 14,000 *g* for 30 min at 4°C to remove insoluble material. The supernatant lysate was subjected to the click reaction with 5 μM Cy5 DBCO for 2 h at room temperature on a rotary shaker. Meanwhile, 20 μl Protein A Agarose beads were incubated in 1 ml of the lipid binding buffer (the same as the lysis buffer) with 1% BSA for 1 h at 4°C on a rotary shaker. Then the beads were washed once with the lipid binding buffer without detergent. The beads were divided into aliquots of equal volume and supplemented with 1 μg of nonspecific rabbit IgG (control) or 1.5 μg of anti-acetylated tubulin rabbit IgG or 4 μg of anti-Arl13b rabbit IgG in 200 μL of 0.1% BSA lipid binding buffer (the same as the lysis buffer) and incubated for 2 h at 4°C on a rotary shaker. The beads were washed once with 1% Triton X-100 lipid binding buffer (the same as the lysis buffer), and lysates from the click reaction were added to the corresponding beads and incubated overnight at 4°C on a rotary shaker. The incubation reaction was centrifuged for 5 min at 1,000 *g*, the supernatant discarded, and the beads were washed five times with 1% Triton X-100 lipid binding buffer (the same as the lysis buffer). The beads were eluted with SDS-sample buffer (Sigma) containing 6 M urea for 30 min at 60°C and then subjected to SDS-PAGE for immunoblotting. The same protocol was adopted when cells were treated with 2-BPA and FB1.

### Visualization of S-palmitoylated proteins in SDS-gels/immunoblots

*C. reinhardtii* or mammalian cells were labeled with 50 μM PAz as described in the previous section and then harvested by centrifugation at 1,000 *g* for 10 min at RT. The resultant pellet was gently washed two times with PBS to remove unreacted fatty acid by centrifugation at 1,000 *g* for 10 min at room temperature. Then, the pellet was fixed by adding ice-cold methanol and incubated for 20 min on ice, washed twice with methanol, and then once with PBS and centrifuged at 1,000 *g* for 10 min at room temperature. The final pellet was lysed in the lipid binding buffer (20 mM Tris–HCl, 150 mM NaCl, 1 mM EDTA, pH 7.5) supplemented with protease/phosphatase inhibitors at 1:100 (Roche) with 1% SDS using sonication. The solubilized protein was centrifuged at 14,000 *g* for 10 min at 4°C to remove insoluble material. The supernatant was subjected to click reaction by incubation with 5 μM Cy5 DBCO (diluted 1:1,000 from 5 mM stock in DMSO) for 4 h at RT on a rotary shaker. Immediately after the click reaction, protein precipitation (chloroform/methanol precipitation) was performed, the pellet briefly dried, and protein solubilized with SDS-sample buffer (Sigma) containing 6 M urea for 30 min at 60°C. Protein was analyzed by SDS-PAGE, protein bands excised for proteomics analysis, or gels further processed for immunoblotting and visualization of palmitoylated proteins.

### Visualization of S-palmitoylated proteins and protein complexes using immunofluorescence microscopy and PLA

*C. reinhardtii* or mammalian cells (HEK293T, human iPSC–derived neurons or primary cultured astrocytes or ependymal cells) grown in suspension or adherent on glass cover slips were labeled with 50 μM PAz as described in one of the previous sections. After fixation with 4% p-formaldehyde/0.5% glutaraldehyde for 15 min at room temperature, cells were subjected to the click reaction (with 5 μM Cy5 DBCO for 1 h at room temperature on a rotary shaker under protection from light). Cells were then permeabilized with 0.2% Triton X-100 in PBS for 10 min at RT, followed by blocking of nonspecific binding sites with 3% ovalbumin in PBS for 1 h at 37°C, and then incubation with primary antibodies for overnight at 4°C as indicated in figure legends at concentrations provided in the Materials and methods section. Then, cells were washed with PLA labeling buffer before performing the PLA reaction following the manufacturer's manual (Duolink) and as described previously (24). Samples were postincubated with secondary antibodies conjugated to fluorophores to detect the subcellular distribution of antigens tested in the PLA assay. Cover slips were embedded using Fluoroshield supplemented with DAPI (Sigma-Aldrich) to visualize the nuclei. Epifluorescence microscopy was performed with a Nikon Ti2 Eclipse microscope equipped with NIS Elements software using Z-scanning with 60× or 100× oil immersion objectives at the step size (0.2–0.3 μm) recommended by the software. Image processing was performed using the NIS-Elements 3D deconvolution program at settings recommended by the software (automated) and PLA signals counted using NIS-Elements and NIH ImageJ software. Images shown in figures are 3D rendered Z-scans. For counting, Z-scan images were collapsed onto one plane and thresholds for pixel size and fluorescence intensity defined based on comparison with nondeconvolved images to represent authentic signals. All of the data were collected by laboratory personnel blinded to the origin of the samples from at least three independent cell cultures using at least five randomly selected areas/culture. Images obtained with secondary antibody only were used as negative controls.

### pacFACer cross-linking of S-palmitoylated proteins

Primary cultures of mouse ependymal cells grown on cover slips in a 24-well dish were labeled overnight with 50 μM PAz as described in one of the previous sections. Cells were then incubated for 30 min with 5 μM of the photoactivatable ceramide analog N-(9-(3-pent-4-ynyl-3-H-diazirin-3-yl)-non-anoyl)-D-erythro-sphingosine (pacFACer, diluted 1:1,000 from a 5 mM stock in ethanol/2% dodecane) at 37°C in a humidified atmosphere containing 5% CO_2_. While being kept in the incubator, cells were UV-irradiated at 365 nm for 15 min with a handheld UV lamp being placed on the cell culture dish with the plastic lid removed. Cells were then washed twice with PBS and fixed with 4% p-formaldehyde/0.5% glutaraldehyde in PBS for 20 min at room temperature. To remove any unincorporated PAz and pacFACer, cells were washed twice for 10 min each with methanol at RT. Cells were placed back in PBS and subjected to two consecutive click chemistry reactions. The first reaction was performed by incubating cells with 5 μM of Alexa Fluor 488-DBCO (diluted 1:1,000 from a 5 mM stock in DMSO) for 1 h at RT to label the azide group of the S-palmitoyl residue using copper-free click chemistry. Cells were washed again with methanol (twice) and then subjected to the second click chemistry reaction to label the alkyne residue of cross-linked pacFACer with 5 μM of Alexa Fluor 647-azide (diluted 1:1,000 from a 5 mM stock in DMSO) using copper-catalyzed click chemistry following the instructions of the manufacturer (Click-IT cell reaction buffer kit from Thermo Fisher) as described previously (1, 16). After the dual click chemistry reactions, immunolabeling using anti-acetylated tubulin mouse IgG was performed as described in one of the previous sections.

### Motility assay

To analyze the phototaxis behavior, *C. reinhardtii* was cultivated at a density of logarithmic growth rate and treated with 50 μM 2BPA overnight and then transferred to a 6-well dish. The wells were shielded from light in such a way that cells were only exposed to light coming from one defined direction. Cells with intact motility move toward and accumulate at the side exposed to light for 0, 10, and 20 min. Cells were counted as described previously (16). The effect of 2BPA on phototaxis motility was observed under the epifluorescence microscope (Nikon Eclipse Ti2 or equivalent epifluorescence) and recorded as images and videos.

### SDS-PAGE and immunoblots

Protein concentrations for pull-down experiments were determined using the RC/DC protein assay (Bio-Rad) following the manufacturer's protocol. For immunoblot analysis, equal amounts of protein were loaded onto a 4%–20% gradient gel, and SDS–PAGE was performed using the Laemmli method. Ponceau S staining was performed to confirm equal loading. The immunoblotting reaction was performed by first blocking the nonspecific binding sites of membranes with 5% nonfat dry milk in PBST (PBS containing 0.1% Tween-20) and incubated with primary antibodies diluted in PBST overnight at 4°C on a rotary shaker. Membranes were then washed three times with PBST and incubated with the appropriate horseradish peroxidase–conjugated secondary antibodies or fluorescent secondary antibody conjugates for 1 h at room temperature on a rotary shaker. After washing, bands were detected using the Pico or Femto ECL assay from Pierce/Thermo Fisher Scientific and Azure imager.

### Statistical analysis

All results shown are representative of at least 3 separate experiments. Statistical analysis was carried out using Student's *t* test with or one-way ANOVA and Tukey's post hoc with GraphPad Prism. Values of at least *P* < 0.05 were considered significant.

## Results

### Ceramide forms a complex with acetylated tubulin and stabilizes microtubules in neuronal processes and cilia

**Fig. 1:**
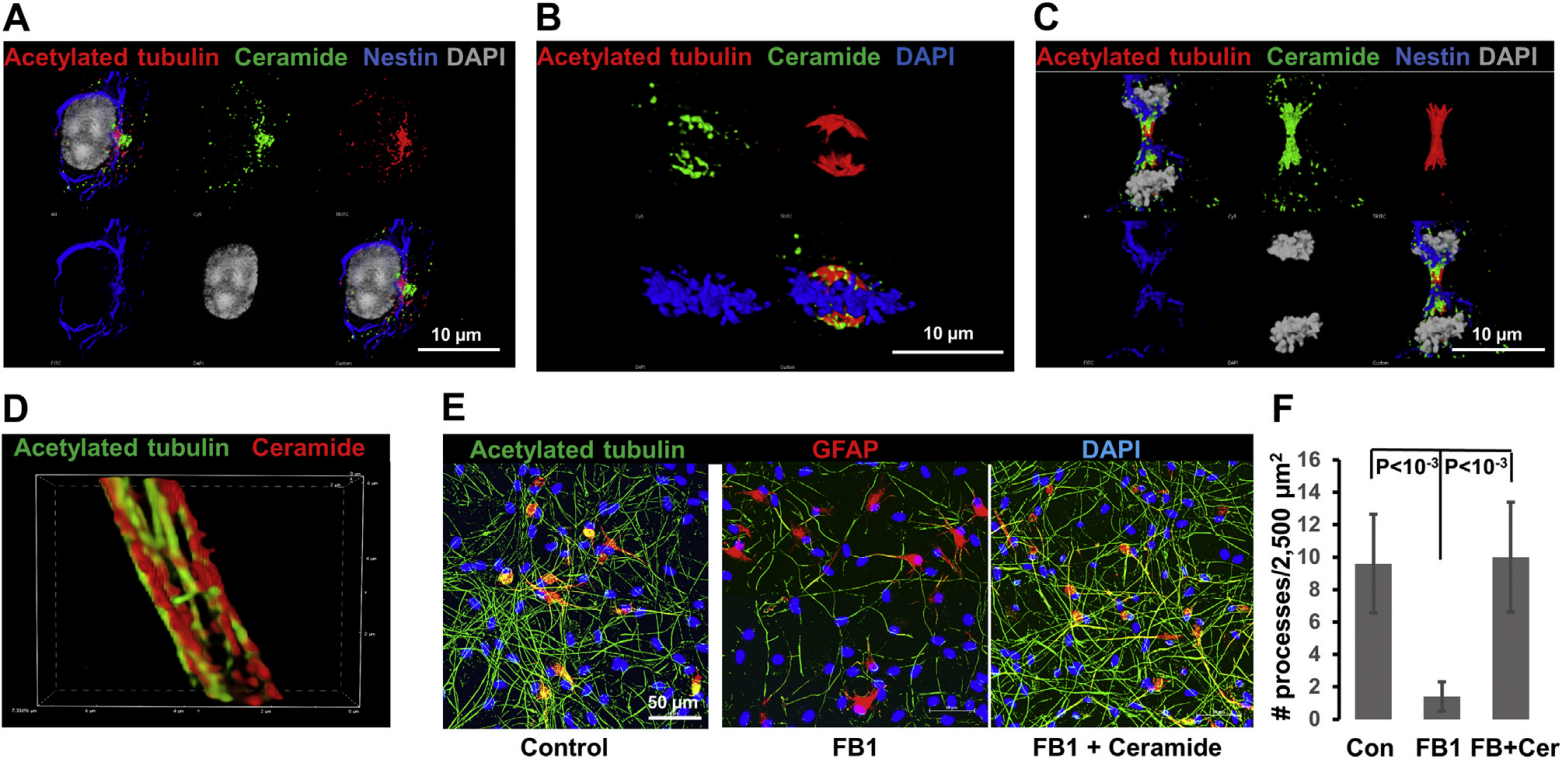
Ceramide colocalizes with acetylated tubulin and stabilizes microtubules in human iPS cell–derived neuroprogenitors and neurons. Human iPS cells were differentiated to neural progenitors (A–C) and then to neurons (D, E). A–C: Immunolabeling with anti-ceramide (green) and anti-acetylated tubulin antibodies (red). In addition, labeling of the neural progenitor marker nestin is shown in (A) and (C) (blue). D: Immunolabeling shows juxtaposition of ceramide (red) to microtubules (acetylated tubulin in green). E, F: Incubation with 10 μM FB1 (middle panel) or 10 μM FB1, and 2 μM C24:1 ceramide (right panel) shows that inhibition of ceramide biosynthesis disrupts neuronal process formation (middle panel), whereas addition of C24:1 ceramide rescues neuronal processes (right panel, the left panel is control). Acetylated tubulin is shown in green. Differentiation is not likely to be affected as shown by expression of the astrocyte marker glial fibrillary acidic protein (red). N = 5. *P* < 0.001. FB1, fumonisin B1; iPS, induced pluripotent stem.

Using immunocytochemistry with anti-ceramide antibody, we found extensive colocalization of ceramide with acetylated tubulin at the microtubule organizer (Fig. 1A), mitotic spindle (Fig. 1B), and midbody of dividing NP cells derived from human iPS cells (Fig. 1C). In NP cell–derived neurons, ceramide-containing structures were associated with microtubules (Fig. 1D). The specificity of anti-ceramide IgG was previously confirmed by colabeling of intracellular structures, including microtubules, with a second, commercially available monoclonal anti-ceramide antibody (MAB014 from Glycobiotech) (24). In neurons, depletion of ceramide by incubation with the ceramide synthase inhibitor FB1 reduced process formation by >80%, which was completely rescued by the addition of C24:1 ceramide (Fig. 1E, F). Labeling for the early neuron marker NeuN and astrocyte marker glial fibrillary acidic protein showed that this was not due to increase in the number of neurons or astrocytes but ceramide promoting neuronal process formation (NeuN labeling not shown). These observations prompted us to investigate a mechanism for the interaction of ceramide with acetylated tubulin that promotes formation or stabilization of microtubules.

**Fig. 2:**
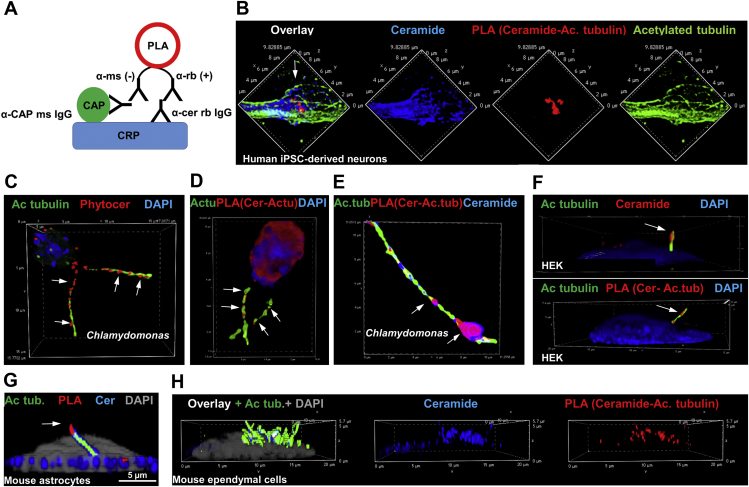
Ceramide forms complexes with acetylated tubulin at microtubules in neuronal processes and cilia. A: PLA for CAPs forming a complex with ceramide at CRPs, the principle of the method. Anti-ceramide rabbit IgG and anti-CAP mouse IgG (here acetylated tubulin) mouse IgG are used for the PLA reaction (red in A–H). Additional immunolabeling with secondary antibodies then shows where the complex is localized within the distribution of ceramide (blue) and acetylated tubulin (green). B: Immunolabeling and PLA using human iPS cell–derived neurons. C–E: The same as in (B) for *Chlamydomonas reinhardtii*. F: Immunolabeling of ceramide and its complex with acetylated tubulin in primary cilia of HEK293T cells. G: The same as in (F) for primary cultured mouse astrocytes. H: The same as in G for primary cultured mouse ependymal cells.

To test direct interaction between ceramide and acetylated tubulin, we developed a novel method for the visualization of complex formation based on the PLA (1). As shown in Fig. 2A, fluorescent signal amplification resulted from a complex of a CAP, here acetylated tubulin, with ceramide, presumably embedded in a CRP (1). Figure 2B shows that the PLA signal colocalized with ceramide and acetylated tubulin in microtubules of neuronal processes. Based on our previous studies and experiments in the present study demonstrating that colocalization of phytoceramide and acetylated tubulin is evolutionarily conserved and already found in flagella of the green algae *C. reinhardtii* (Fig. 2C) (16), we tested if flagella contain phytoceramide-acetylated tubulin complexes. Anti-ceramide rabbit IgG detected ceramide and phytoceramide as confirmed in our previous studies (16, 27). Figure 2D and E shows that phytoceramide labeling was colocalized with PLA signals for its complex with acetylated tubulin (arrows), indicating that CRPs in the ciliary membrane engage into complexes with acetylated tubulin. Likewise, ceramide-acetylated tubulin complexes were found in primary cilia of human embryonic kidney 293T cells (Fig. 2F, arrows) and primary cultured mouse astrocytes (Fig. 2G, arrow), and motile cilia of primary cultured mouse ependymal cells (Fig. 2H, arrows). These data show that ceramide forms a complex with acetylated tubulin and that this complex is likely to contribute to formation or stabilization of microtubules in cilia.

### S-palmitoylation of acetylated tubulin in *C. reinhardtii* is critical for motile cilium function

**Fig. 3:**
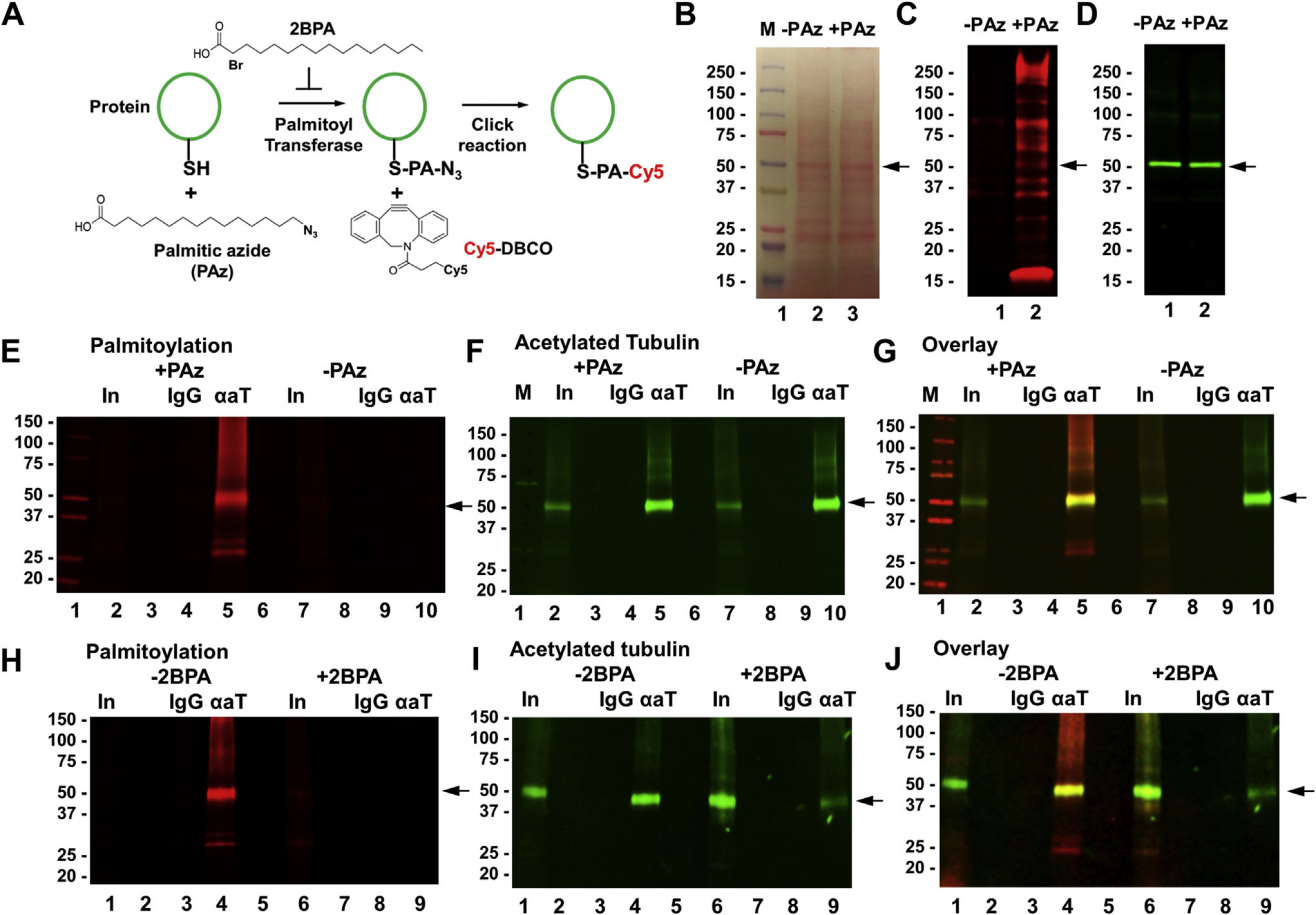
Acetylated tubulin is S-palmitoylated in *Chlamydomonas reinhardtii.* A: Metabolic labeling of S-palmitoylated proteins with palmitic azide (PAz), the principle of the method. Cells (here *C. reinhardtii*) are metabolically labeled with PAz and then subjected to click reaction–mediated addition of a fluorophore (here Cy5) to the S-palmitoylated protein. 2-Bromo palmitic acid (2BPA) is used to specifically inhibit S-palmitoylation. B–D: SDS-PAGE and immunoblot of protein from *C. reinhardtii* metabolically labeled with PAz and subjected to click reaction as described in (A). B: Ponceau staining; (C) Cy5 fluorescence of S-palmitoylated protein (pseudocolored in red); (D) immunoblot for acetylated tubulin (pseudocolored in green). −PAz is the negative control without PAz. E–J: Immunoprecipitation of S-palmitoylated protein using anti-acetylated tubulin rabbit IgG for the precipitation reaction and anti-acetylated tubulin mouse IgG for immunoblotting. −PAz is the negative control without S-palmitoylation. E–G: Show immunoblot of the precipitate with metabolic PAz labeling, but without inhibition of S-palmitoylation. H–J: The same as in (E–G), but with inhibition of S-palmitoylation (2BPA). αaT, precipitation reaction with anti-acetylated tubulin rabbit IgG; IgG, precipitation control with nonspecific rabbit IgG; In, input.

Complex formation of membrane lipids with proteins is mediated by either direct interaction or anchoring of acyl residues such as palmitoyl residues reversibly linked to cysteines in the protein (S-palmitoylation) bound to the membrane. Because affinity for direct interaction between ceramide and tubulin is rather low (24) and tubulin can be S-palmitoylated (23), we tested if acetylated tubulin is anchored to ceramide via S-palmitoyl residues, particularly in cilia. We investigated this interaction using a method recently introduced for detecting S-palmitoylated proteins (23). As shown in Fig. 3A, HEK293T cells were fed with PAz, which is incorporated into S-palmitoylated proteins. Click chemistry–mediated addition of a fluorophore (here Cy5 DBCO) to the azide group then labeled the S-palmitoylated proteins for analysis by SDS-PAGE/blotting and fluorescence microcopy. Inhibition of labeling by the palmitoyl transferase inhibitor 2BPA confirmed specificity of metabolic labeling with PAz and click chemistry–mediated addition of the fluorophore. We used this method for the first time to detect S-palmitoylated proteins in *C. reinhardtii*. Figure 3B–D shows that when separating and immunoblotting equal amounts of protein from *C. reinhardtii* (Ponceau staining in Fig. 3B), only protein from algae fed with PAz showed fluorescent bands indicative of S-palmitoylation (Fig. 3C, lane 2), whereas proteins from algae not fed with PAz were not fluorescent (Fig. 3C, lane 1). This result showed that *C. reinhardtii* incorporated PAz into protein that was specifically detected by covalently linking Cy5 DBCO using click chemistry. We also found that one of the S-palmitoylated protein bands colabeled with acetylated tubulin (Fig. 3D), which suggested that acetylated tubulin was S-palmitoylated.

To confirm that acetylated tubulin was S-palmitoylated and that the palmitoylation reaction was catalyzed by palmitoyl transferase, we performed immunoprecipitation of acetylated tubulin isolated from *C. reinhardtii* cells that were metabolically labeled with PAz in the presence or absence of 2BPA. Using equal amounts of input protein (lanes 2 and 7 in Fig. 3E–G) and antiacetylated tubulin antibody, we showed that immunoprecipitated acetylated tubulin (lanes 5 and 10 in Fig. 3F, G) was also S-palmitoylated (lane 5 in Fig. 3E, G). Without metabolic labeling with PAz, no fluorescent band was detected (lane 10 in Fig. 3E, G), ruling out nonspecific linking of the fluorophore. Control IgG did not show any fluorescent band (lanes 4 and 9 in Fig. 3E–G) confirming specificity of the immunoprecipitation reaction. When 2BPA was added to PAz, the fluorescence signal for S-palmitoylation disappeared (Fig. 3H–J), confirming that acetylated tubulin was specifically S-palmitoylated by palmitoyl transferase.

**Fig. 4:**
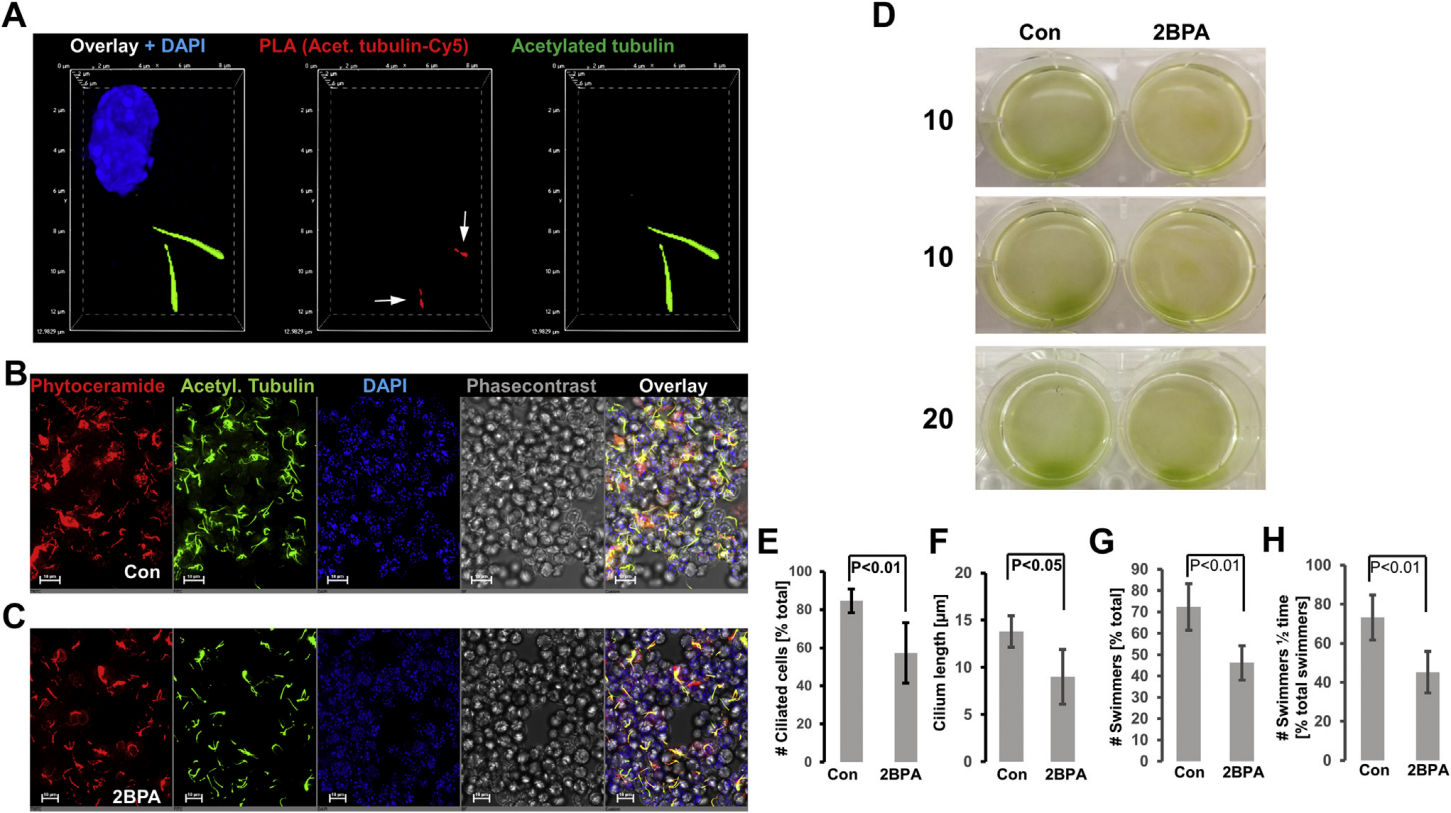
S-palmitoylation of acetylated tubulin in flagella is critical for *Chlamydomonas reinhardtii* motility. A: PLA reaction for S-palmitoylated acetylated tubulin shows signals in the flagella (red, arrows). Acetylated tubulin is shown in green. B, C: Inhibition of S-palmitoylation with 2BPA shows reduction in the number of flagella (acetylated tubulin, green; ceramide, red). D: Motility assay. 2BPA impairs swimming velocity. E–H: Quantitation of (B–D). E: Shows the number of ciliated *C. reinhardtii*, F shows the average flagella length, (G) shows the proportion of cells that swim toward the direction of the light source in (D) after 20 min, and (H) shows the proportion of swimmers that reach the end of the well after half-time (10 min in D). N = 5. *P* as indicated in figure.

Next, we determined the subcellular distribution of S-palmitoylated and acetylated tubulin combining the click chemistry–based palmitoylation assay with PLA in immunocytochemistry. To visualize palmitoylated and acetylated tubulin, the PLA was performed with antibodies against acetylated tubulin and Cy5 covalently bound to the incorporated PAz using click chemistry (1, 23). Figure 4A shows that S-palmitoylated and acetylated tubulin was located at the tip of flagella (arrows). The PLA signals were not detected when *C. reinhardtii* was treated with 2BPA (not shown). 2BPA treatment reduced the number and length of flagella by about 40% (Fig. 4B, C, E, F), concomitant with the decreased number of swimmers and motility (Fig. 4D, G, H, and supplemental Movies 1 and 2). These data showed that S-palmitoylation of acetylated tubulin is critical for formation of flagella and motility.

### S-palmitoylation is necessary for interaction of acetylated tubulin with Arl13b and ciliogenesis in mammalian cells

**Fig. 5:**
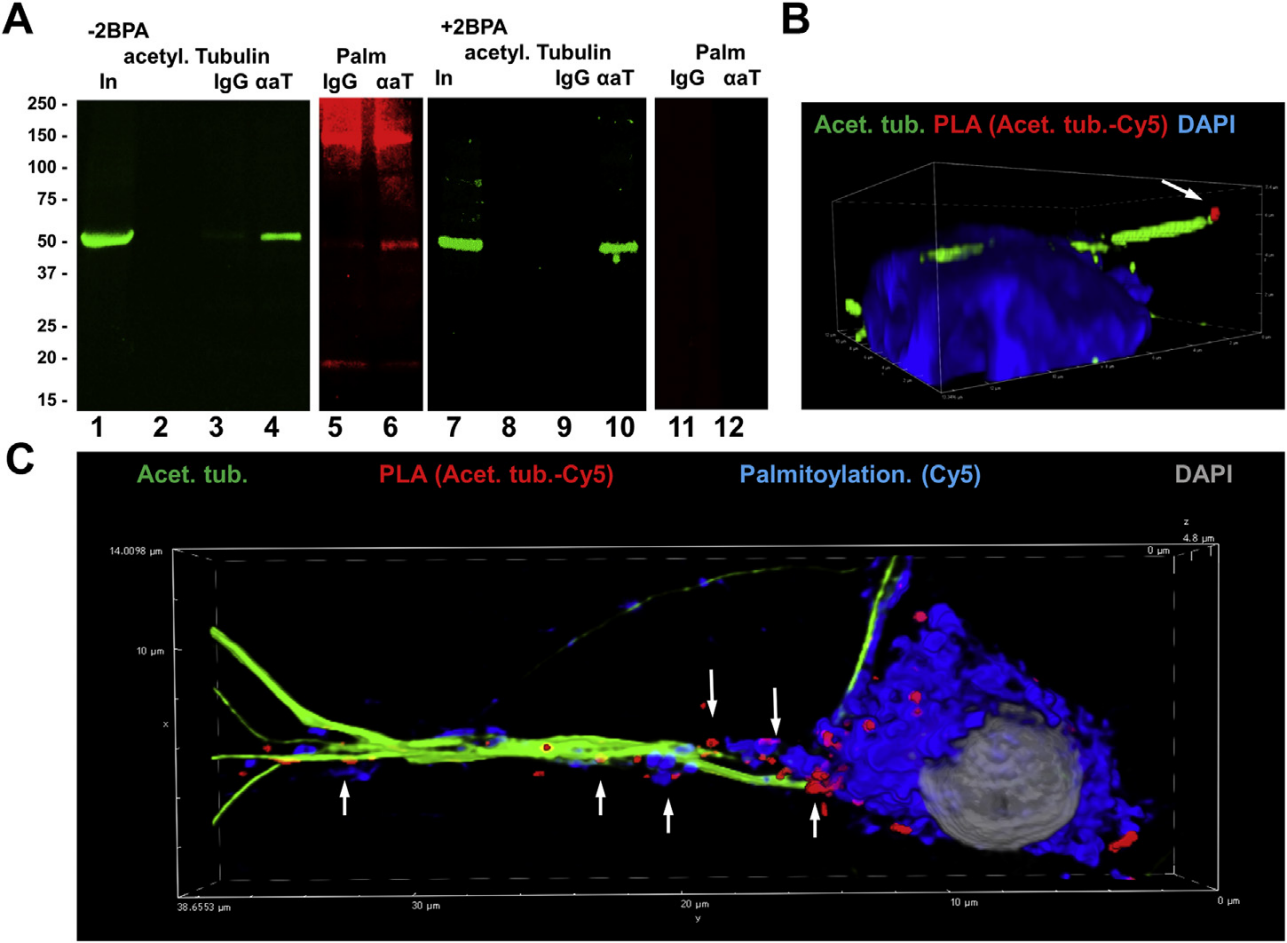
Acetylated tubulin in microtubules of mammalian cells is S-palmitoylated. A: Metabolic labeling of HEK293T cells with PAz, with or without inhibition of S-palmitoylation with 2BPA, followed by click chemistry–mediated addition of fluorophore (Cy5) and immunoprecipitation using anti-acetylated tubulin rabbit IgG. Immunoblot developed with anti-acetylated tubulin mouse IgG (pseudocolored in green). Palmitoylation is pseudocolored in red. B, C: PLA reaction for S-palmitoylation of acetylated tubulin shows signal at primary cilia of HEK293T cells (B, arrow) and along microtubules of neuronal processes in human iPS cell–derived neurons (C, arrows). Acetylated tubulin labeling is shown in green and S-palmitoylation in blue (C). αaT, immunoprecipitation using anti-acetylated tubulin rabbit IgG; 2BPA, 2-bromo palmitic acid; IgG, control immunoprecipitation using nonspecific rabbit IgG; In, input; iPS, induced pluripotent stem; PAz, palmitic azide; PLA, proximity ligation assay.

Our data showing that acetylated tubulin is S-palmitoylated in *C. reinhardtii* prompted us to perform similar experiments in mammalian cells. We used HEK293T cells for incubation with PAz, click chemistry–mediated addition of Cy5, and immunoprecipitation reactions. Figure 5A shows that immunoprecipitated acetylated tubulin was S-palmitoylated, which was prevented by 2BPA. Similar to results obtained with *C. reinhardtii*, PLA showed that S-palmitoylated and acetylated tubulin was transported to cilia (Fig. 5B). In human iPS cell–derived neurons, acetylated tubulin was also S-palmitoylated at microtubules in neuronal processes that colabeled for compartments with S-palmitoylated proteins (Fig. 5C), suggesting that S-palmitoylated acetylated tubulin was associated with other S-palmitoylated proteins in membrane contact sites to the cytoskeleton (arrows).

**Fig. 6:**
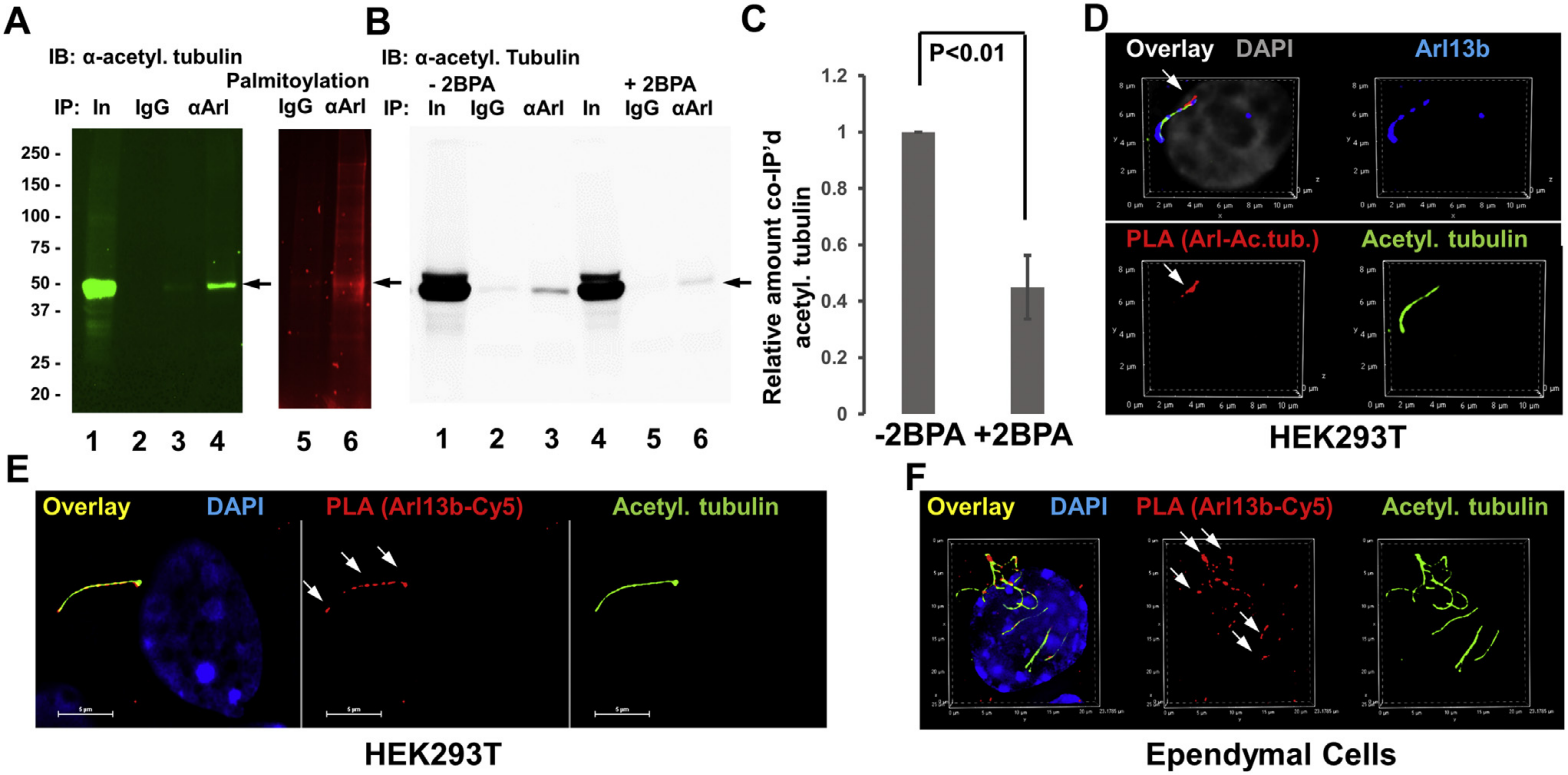
Acetylated tubulin forms a complex with S-palmitoylated Arl13b in cilia. A–C: Metabolic labeling of HEK293T cells with PAz, followed by click chemistry–mediated addition of fluorophore (Cy5) and immunoprecipitation using anti-Arl13b rabbit IgG. Immunoblot shows coimmunoprecipitated acetylated tubulin, the signal of which is reduced by 2BPA (arrow in B). Quantitation is shown in C. N = 3. *P* < 0.01. D: PLA for complex of Arl13b with acetylated tubulin (signal in red, arrow) in HEK293T cells. Acetylated tubulin labeling is shown in green and Arl13b in blue. E, F: PLA for S-palmitoylation of Arl13b in HEK cells (E) and ependymal cells (F). Arrows point at S-palmitoylated Arl13b (red) in cilia. Acetylated tubulin labeling is shown in green. αArl, immunoprecipitation using anti-Arl13b rabbit IgG (immunolabeled Arl13b is pseudocolored in green, and palmitoylation is pseudocolored in red); 2BPA, 2-bromo palmitic acid; IgG, control immunoprecipitation using nonspecific rabbit IgG; In, input; PAz, palmitic azide; PLA, proximity ligation assay.

**Fig. 7:**
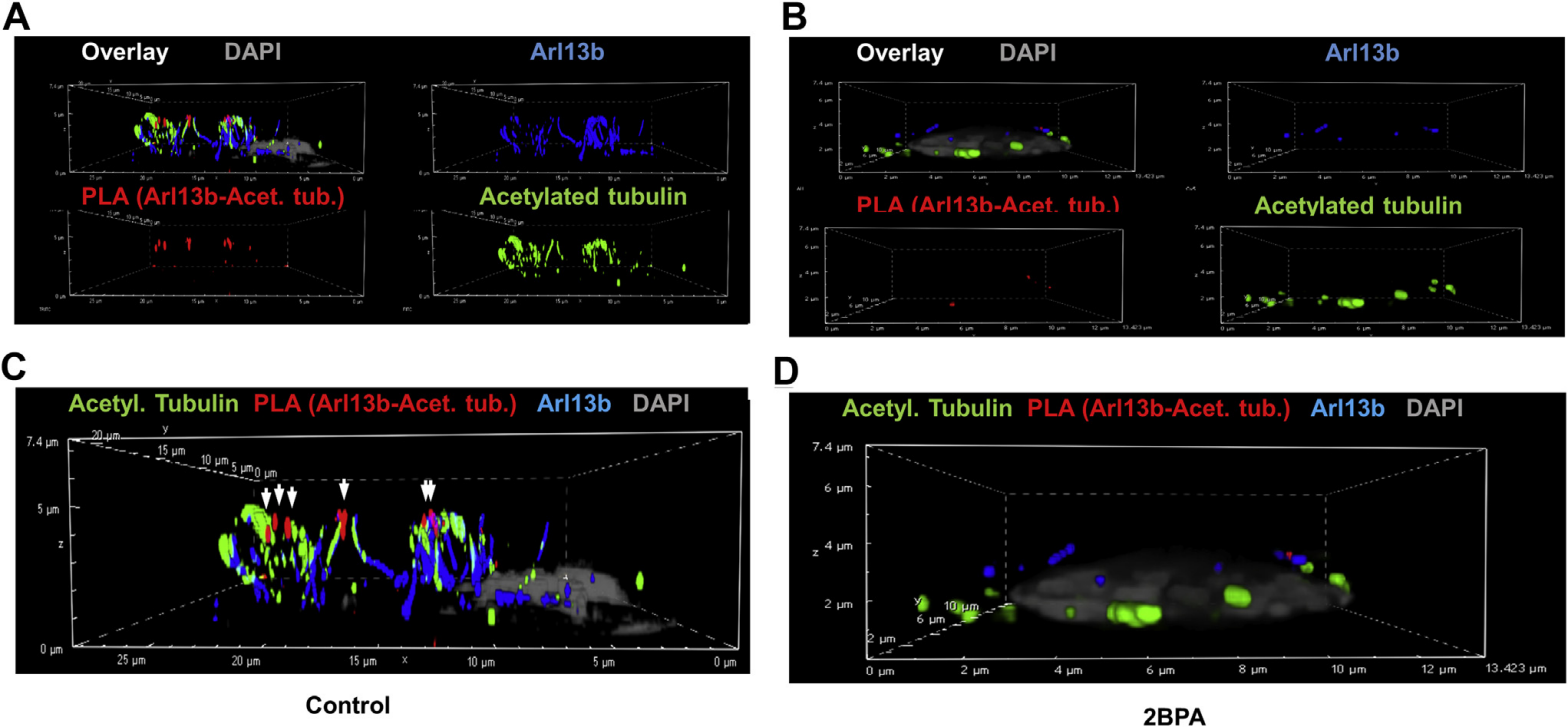
S-palmitoylation is critical for complex formation of acetylated tubulin with Arl13b and ciliogenesis. A–-D: PLA for complex of Arl13b with acetylated tubulin (signals pseudocolored in red, arrows) in primary culture of mouse ependymal cells, with or without inhibition of S-palmitoylation (2BPA). Labeling of acetylated tubulin is shown in green and Arl13b is in blue. A, C: (detail of A) without 2BPA; B and D (detail of B) with 2BPA showing that inhibition of S-palmitoylation disrupts Arl13b–acetylated tubulin complex formation and ciliogenesis. 2BPA, 2-bromo palmitic acid; PLA, proximity ligation assay.

Tubulin is known to bind to Arl13b, an S-palmitoylated GTPase that transports proteins into cilia (18, 19, 28). Using coimmunoprecipitation assays and PLAs, we tested if S-palmitoylated and acetylated tubulin was associated with Arl13b. Figure 6A shows that acetylated tubulin coimmunoprecipitated with anti-Arl13b rabbit IgG was also S-palmitoylated (arrow). Inhibition of S-palmitoylation with 2BPA reduced complex formation by more than 50% (Fig. 6B, C), indicating that S-palmitoylation of acetylated tubulin and/or Arl13b is critical for interaction of acetylated tubulin with Arl13b. Complex formation between acetylated tubulin and Arl13b was confirmed by PLAs (Fig. 6D, arrow). The combined S-palmitoylation/PLA reaction showed that ciliary Arl13b was S-palmitoylated in primary and motile cilia of HEK293T cells and ependymal cells, respectively (Fig. 6E, F). Incubation of ependymal cells with 2BPA obliterated formation of motile cilia (Fig. 7A–D), demonstrating that S-palmitoylation was critical for cilium formation. Interestingly, PLA signals in ependymal cell motile cilia were mainly located at the tip of cilia (arrows in Fig. 7D), suggesting that complexes between acetylated tubulin and Arl13b mediated transport of acetylated tubulin and that inhibition of S-palmitoylation interferes with this transport.

### Ceramide mediates interaction and ciliary transport of S-palmitoylated proteins

**Fig. 8:**
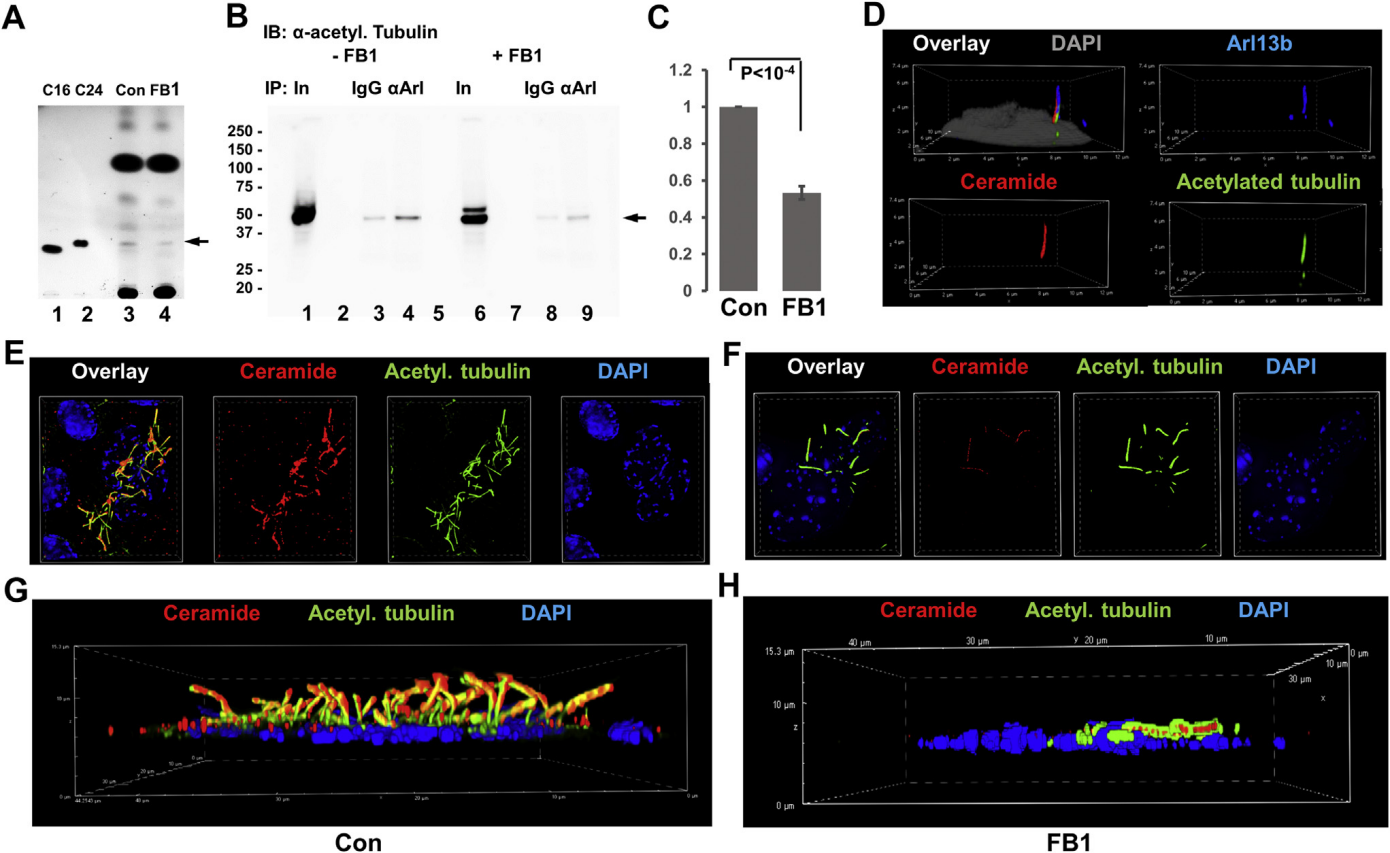
Ceramide is critical for complex formation of acetylated tubulin with Arl13b and formation of cilia. A: High-performance thin-layer chromatography (HPTLC) for ceramide isolated from control and FB1-treated HEK293T cells. The arrow points at the ceramide band, the intensity of which is reduced by FB1-mediated inhibition of ceramide biosynthesis. C16, C24, ceramide standards C16:0 ceramide and C24:1 ceramide. B, C: Coimmunoprecipitation assay using anti-Arl13b rabbit IgG for the precipitation reaction and acetylated tubulin mouse IgG for immunoblotting. Incubation with FB1 shows that reduction of ceramide levels as shown in (A) leads to lower amount of coimmunoprecipitated acetylated tubulin (arrow). Quantitation is shown in (C). N = 3. *P* < 0.0001. D: Immunocytochemistry shows colocalization of acetylated tubulin (green), ceramide (red), and Arl13b (blue) in primary cilia of HEK293T cells. E–H: Immunocytochemistry for acetylated tubulin (green) and ceramide (red) shows that inhibition of ceramide biosynthesis with FB1 disrupts ciliogenesis in primary cultured mouse ependymal cells. FB1, fumonisin B1.

**Fig. 9:**
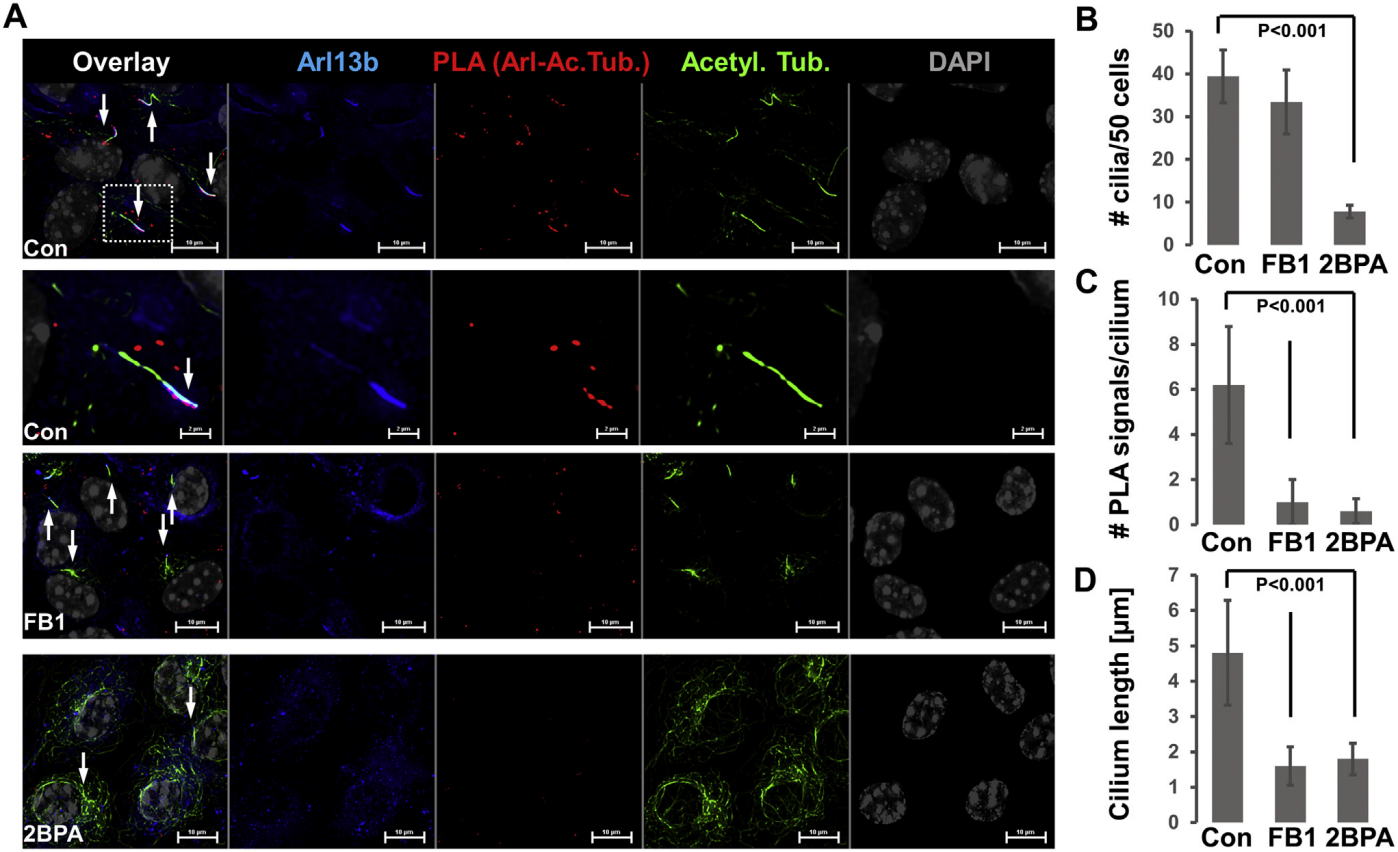
FB1 and 2BPA prevent complex formation between acetylated tubulin and Arl13b in primary cilia of astrocytes. A: PLA detecting Arl13b-acetylated tubulin complexes (red) and immunocytochemistry for Arl13b (blue) and acetylated tubulin (green) shows that reduction of ceramide levels with FB1 (48 h, 10 μM) or 2BPA (16 h, 10 μM) disrupts complex formation and ciliogenesis in primary cultured mouse astrocytes. Arrows point at cilia. Second panel from the top shows magnified area from the top panel (control). Arrows point at Arl13b-acetylated tubulin complexes that are mainly detected at the top part of primary cilia. B–D: Quantitation of the number of cilia in randomly chosen areas of 50 cells (B), the number of PLA signals/cilium (C), and cilium length (D). N = 5. *P* as indicated in figure. FB1, fumonisin B1; PLA, proximity ligation assay.

**Fig. 10:**
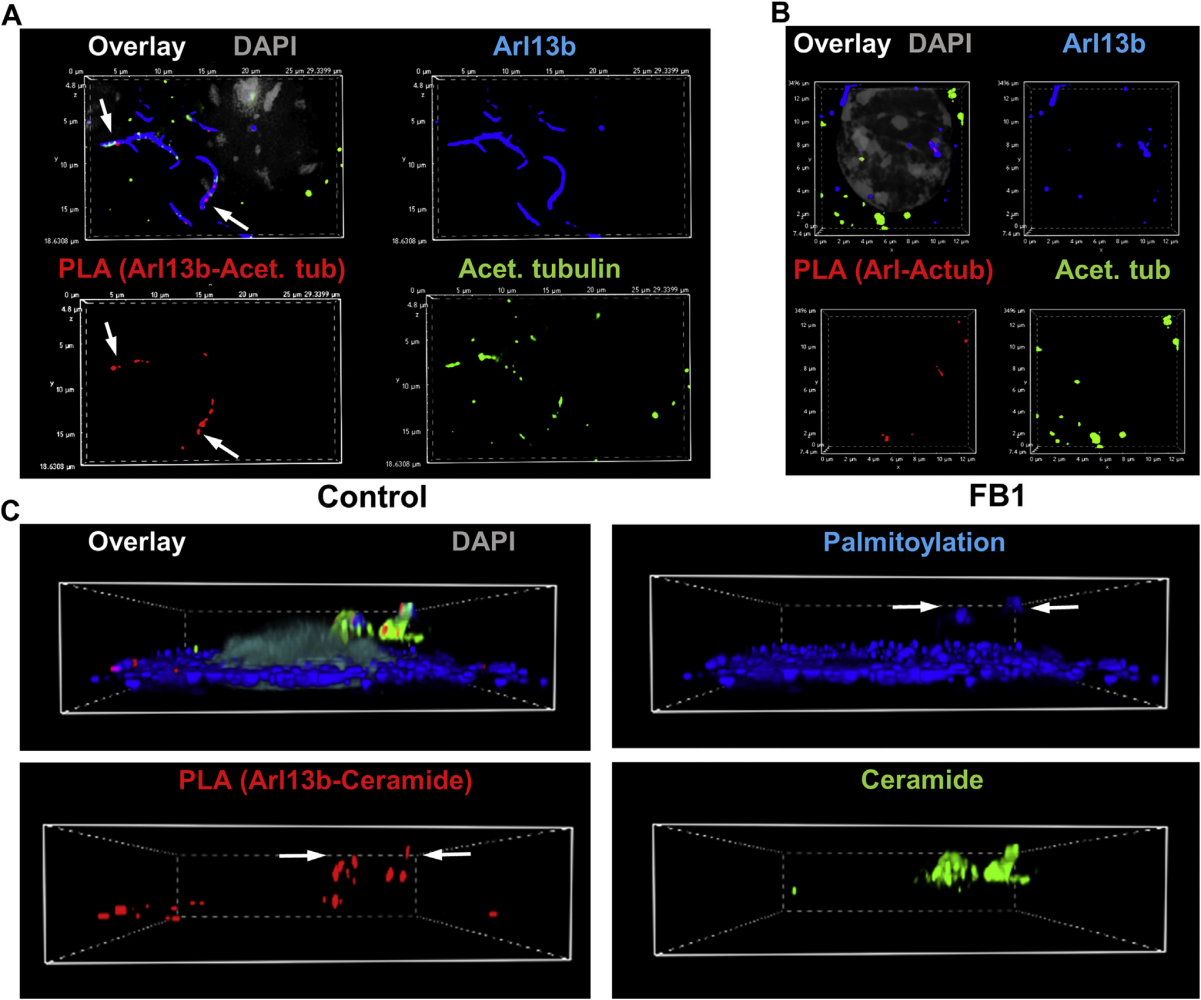
Ceramide associates with Arl13b, which is critical for complex formation with acetylated tubulin in motile cilia of ependymal cells. A, B: The PLA detecting Arl13b-acetylated tubulin complexes (red) and immunocytochemistry for Arl13b (blue) and acetylated tubulin (green) shows that reduction of ceramide levels with FB1 disrupts complex formation and ciliogenesis in primary cultured mouse ependymal cells (arrows). C: Ependymal cells were metabolically labeled with PAz and S-palmitoylation visualized by click chemistry–mediated addition of fluorophore (Cy5). The PLA was performed using antibodies against ceramide and Arl13b showing that S-palmitoylation (blue) is at least partially colocalized with ceramide-acetylated tubulin complexes (red, arrows). Ceramide immunolabeling is shown in green. FB1, fumonisin B1; PAz, palmitic azide; PLA, proximity ligation assay.

Our data showed that acetylated and S-palmitoylated tubulin interacted with ceramide and formed a protein complex with S-palmitoylated Arl13b, suggesting that formation of the complex was mediated by ceramide in the ciliary membrane. This hypothesis was in line with previous studies showing that S-palmitoylated proteins associated with lipid rafts in the plasma membrane, particularly when enriched with ceramide (12). To test ceramide-mediated complex formation between Arl13b and acetylated tubulin, we performed coimmunoprecipitation assays in the absence and presence of FB1, an inhibitor for ceramide generation by ceramide synthases. Using high-performance thin-layer chromatography, we confirmed that incubation of HEK293T cells with 10 μM FB1 for 48 h reduced ceramide levels by more than 50% (Fig. 8A, arrow). This reduction of the ceramide level was associated with a decrease in the amount of acetylated tubulin pulled down with anti-Arl13b antibody by 50% (Fig. 8B, C, lanes 4 and 9, arrow), indicating that ceramide mediated complex formation between the two proteins. This conclusion was supported by immunocytochemistry showing that ceramide colocalized with acetylated tubulin and Arl13b in primary cilia of HEK293T cells (Fig. 8D) and motile cilia in ependymal cells (not shown). Inhibition of ceramide biosynthesis with FB1 dramatically reduced the amount of ceramide in motile cilia, concurrent with overall reduction of the number of motile cilia in ependymal cells (Fig. 8E–H). Interestingly, in astrocytes, FB1 did not significantly reduce the number of primary cilia (Fig. 9A, B), but it decreased the number of complexes between acetylated tubulin and Arl13b in cilia by >80% (Fig. 9A, C), concomitant with reduction of the cilium length (Fig. 9A, D). 2BPA, on the other hand, reduced the number of ciliated cells by >80% (Fig. 9A, B), concomitant with reduced complex formation between acetylated tubulin and Arl13b (Fig. 9A, C) and decreased length of the residual cilia (Fig. 9A, D). Noteworthy, formation of the complexes between acetylated tubulin and Arl13b was mainly found in the upper part and tips of primary cilia (arrows in Fig. 9A), which was observed for motile cilia and prevented by FB1 as well (arrows in Fig. 7C, Fig. 10A, B). This observation indicates that the complex formation between acetylated tubulin and Arl13b is critical for cilium elongation at the tip.

**Fig. 11:**
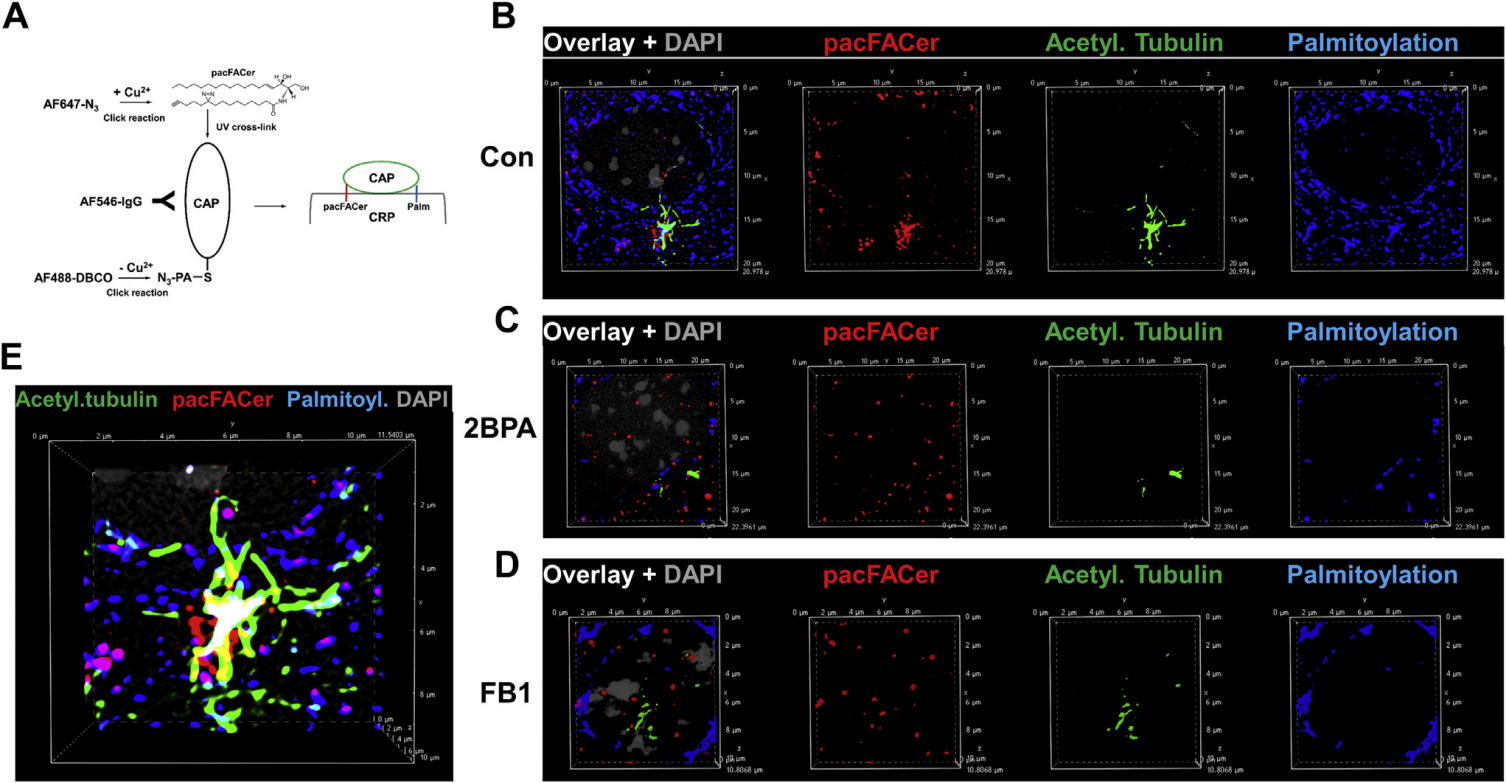
S-palmitoylated protein cross-links to photoactivatable ceramide in cilia, which is prevented by 2BPA and FB1. A: Simultaneous labeling for S-palmitoylation and cross-linking to photoactivatable ceramide (pacFACer), the principle of the method. Cells were incubated with PAz to metabolically label for S-palmitoylation, and then with pacFACer followed by UV cross-linking to label for ceramide binding of the CAP (here acetylated tubulin). The protein-bound S-palmitoyl azide and pacFACer residues were linked to fluorophores using two different click reactions (copper-free for adding fluorophore [AF488-DBCO] to S-palmitoyl azide residue and copper-catalyzed for adding fluorophore [AF647-azide] to pacFACer residue). B–F: Reaction as described for (A) performed with primary cultured mouse ependymal cells and immunocytochemistry using anti-acetylated tubulin mouse IgG shows colocalization of pacFACer-cross-linked protein with S-palmitoylation in cilia (C and E, arrows), unless S-palmitoylation is inhibited with 2BPA (C) or ceramide biosynthesis with FB1 (D). 2BPA, 2-bromo palmitic acid; CAP, ceramide-associated protein; FB1, fumonisin B1.

Next, using S-palmitoylation assay and PLAs in immunocytochemistry with anti-ceramide antibody, we showed that complexes of Arl13b and acetylated tubulin were colocalized with S-palmitoylated proteins and ceramide in motile cilia (Fig. 10C, arrows). These data suggested that ceramide interacted with S-palmitoylated proteins in cilia. To further test the function of ceramide and S-palmitoylation for distribution of ciliary proteins, we used cross-linking to a photoactivatable ceramide analog (pacFACer), a method previously introduced in our laboratory, to visualize the transport of ceramide-associated proteins to cilia (1, 16, 20). pacFACer was cross-linked to proteins that bind to ceramide, and after transport to cilia, the cross-linked proteins were visualized by click chemistry–mediated addition of a fluorophore (Fig. 11A). Cross-linking to pacFACer was combined with PAz-mediated S-palmitoylation and click chemistry–mediated addition of a different fluorophore. Figure 11B and E shows that S-palmitoylated proteins cross-linked to pacFACer and colocalized with acetylated tubulin, indicating that S-palmitoylated proteins were associated with ceramide in the ciliary membrane. This association was disrupted by inhibition of S-palmitoylation with 2BPA and ceramide biosynthesis with FB1 (Fig. 11C, D). We previously showed that ceramide depletion with FB1 reduced the amount of paFACer cross-linked protein because of decreased formation of CRPs that incorporate the ceramide analog before its cross-linking to the CAP (1). Therefore, our results suggest that both S-palmitoylation and ceramide (in CRPs) were critical for transport and interaction of a variety of proteins in cilia.

## Discussion

Our previous studies showed that ceramide critically regulated cilium length and ciliary transport, but the mechanism of this regulation remained elusive. Direct interaction of ceramide with proteins such as atypical protein kinase Cζ/λ, glycogen synthase kinase-3β, and tubulin was detected; however, binding constants were relatively high and unlikely to enable transport of these proteins along cilia (14, 16). In the present study, we focused on tubulin because this protein is mass-transported from the cytosol to the cilium tip to elongate cilia. Furthermore, transport of tubulin in cilia has to be synchronized with adding new ciliary membrane to allow for cilium elongation. Based on our data showing that ceramide is enriched in the ciliary membrane, we hypothesized that ceramide and tubulin are cotransported to the cilium tip, provided that binding between the two can be enhanced.

Tubulin is a protein that shuttles between the cytosol, microtubules, and cellular membranes. Most recently, S-palmitoylated tubulin was found to be distributed along microtubules and associated with the plasma membrane (23). S-palmitoylated proteins are known to associate with lipid rafts in the plasma membrane, particularly when enriched with ceramide (12). Therefore, we tested if S-palmitoylation of acetylated tubulin enhanced its interaction with ceramide in the plasma membrane and other membrane compartments, particularly those interacting with microtubules. Our data showing colocalization and complex formation of acetylated tubulin with ceramide at the mitotic spindle, midbody, neuronal processes, and cilia (Figs. 1, 2) suggested that ceramide is critical for microtubule assembly and/or stability. Noteworthy, the interaction of ceramide with acetylated tubulin appeared to be increased at microtubule repair sites. This assumption was confirmed by the observation that inhibition of ceramide biosynthesis with FB1 disrupted neuronal processes (Fig. 1D) and cilia (Figs. 8, 9), while processes were restored by the addition of C24:1 ceramide (Fig. 1E, F). We previously showed that the same ceramide species was taken up by iPS cell–derived neuroprogenitors and promoted cilium elongation, although the exact mechanism for this remained unclear (14, 16). These studies invoked C24:1 ceramide binding to glycogen synthase kinase-3β, a protein kinase we showed to interact with phytoceramide in *C. reinhardtii* flagella and ceramide in mammalian cilia. Based on these data, we assumed that S-palmitoylation of acetylated tubulin and its interaction with phytoceramide and ceramide may be another mechanism underlying regulation of cilia that is evolutionarily conserved from *C. reinhardtii* to mammalian cells.

There is only little information on S-palmitoylation of proteins in *C. reinhardtii*. In one study, metabolic labeling with radioactive palmitic acid showed several proteins in the molecular mass range of tubulin, however, without being further identified (29). We introduced metabolic labeling with PAz followed by click chemistry–mediated fluorophore addition and immunoprecipitation assays or PLAs as novel approaches to determine S-palmitoylation of acetylated tubulin in *C. reinhardtii* (Figs. 3, 4) and mammalian cells (Figs. 5, 6). To our knowledge, our study is the first to identify S-palmitoylation of acetylated tubulin in *C. reinhardtii*. Furthermore, for the first time, we show that inhibition of S-palmitoylation with 2BPA disrupts formation of flagella and impairs ciliogenesis in mammalian cells (Figs. 4, 6, 9), suggesting that S-palmitoylation of acetylated tubulin is critical for the function of microtubules in cilia and other compartments. Our observations are in line with other studies showing that mutation of S-palmitoylation sites in tubulin or incubation with 2BPA leads to abnormal mitotic spindle formation and impairment of neurite outgrowth in yeast and dorsal root ganglion neurons, respectively (17, 30).

Owing to the observation that depletion of ceramide and inhibition of S-palmitoylation disrupted cilia, we tested the hypothesis that interaction of the S-palmitoyl residue with ceramide mediated ciliary transport of S-palmitoylated proteins, including acetylated tubulin and Arl13b. Arl13b binds to acetylated tubulin to uniformly distribute it along the ciliary membrane. S-palmitoylation of Arl13b is critical for this transport function (19, 28). It is not known if S-palmitoylated Arl13b interacts with palmitoylated acetylated tubulin and whether this heterocomplex is anchored to ceramide in the ciliary membrane via the S-palmitoyl residues. Because 2BPA and FB1 reduce complex formation between Arl13b and acetylated tubulin (Fig. 7, Fig. 8, Fig. 9, Fig. 10, Fig. 11), we conclude that the two proteins are anchored to ceramide via their S-palmitoyl residues, which is followed by complex formation in the ciliary membrane. This conclusion is also supported by the observation that protein cross-linked to a ceramide analog (pacFACer) was distributed to cilia, unless palmitoylation or ceramide biosynthesis was inhibited (Fig. 11).

**Fig. 12:**
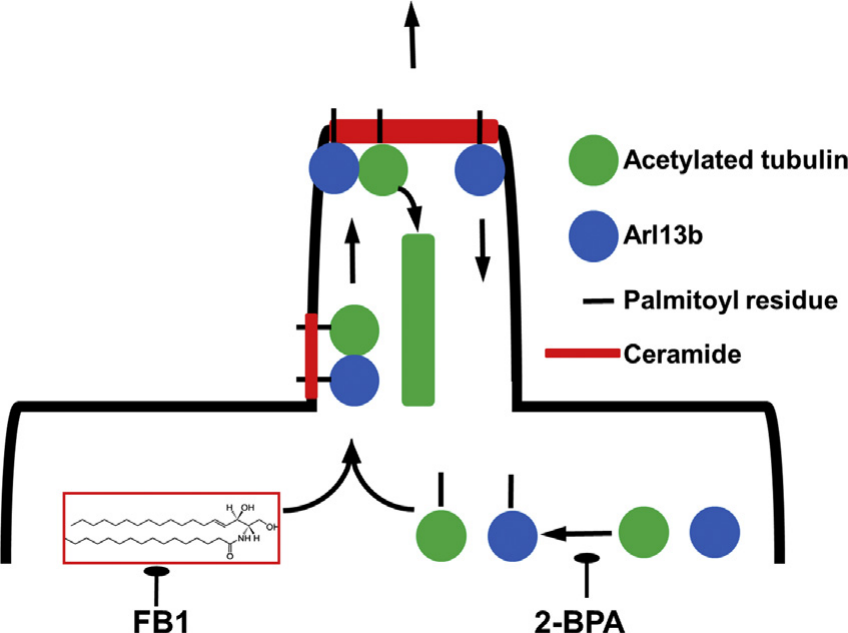
Regulation of ciliary protein complex formation by S-palmitoylation and association with ceramide. In this model, Arl13b and acetylated tubulin are S-palmitoylated and then bound to ceramide in the ciliary membrane at the cilium base. The two proteins form a complex that is transported with CRPs to the cilium tip. Acetylated tubulin is used to extend the growing axoneme, and Arl13b is transported back to the base. Depletion of ceramide with FB1 or inhibition of S-palmitoylation with 2BPA will disrupt binding of Arl13b and acetylated tubulin to the ciliary membrane and subsequently complex formation and transport of the two proteins to the cilium tip. 2BPA, 2-bromo palmitic acid; CRP, ceramide-rich platform; 2FB1, fumonisin B1.

Figure 12 depicts the function of S-palmitoylation and anchoring to ceramide for the transport of ciliary proteins. In the first step, cytosolic proteins such as Arl13b and acetylated tubulin are S-palmitoylated, which mediates attachment to the membrane. In the second step, the two proteins are translocated to the same membrane microdomains or rafts such as CRPs where they come sufficiently close to form dimers. In the third step, these dimers are cotransported with CRPs to the tip of the cilium. Acetylated tubulin is then incorporated into the nascent microtubules of the cilium, whereas Arl13b is transported back to the cilium base.

Although the present study focuses on the function of S-palmitoylation of Arl13b and acetylated tubulin, this proposed mechanism of CRP-mediated protein complex formation and transport could be generalized to other S-palmitoylated proteins and lipid rafts such polycystin in cilia and Rab proteins in vesicular transport (31). Recently, another study found that ceramide-rich lipid rafts (equivalent to CRPs) are critical for transport of >100 palmitoylated proteins from Golgi to the plasma membrane, although the function of this interaction for complex and ciliary transport was not investigated (12). It is currently unclear why the palmitoyl residue shows specific affinity to ceramide in rafts or platforms. A probable cue comes from the effect of leaflet asymmetry due to C24:1 ceramide, which is also effective in promoting neuronal process formation (Fig. 1C) and cilium extension (14, 16). According to the “leaflet interdigitation model” the overhanging portion of the nervonyl or nervonoyl chain in C24:0 or C24:1 ceramide protrudes into the adjacent membrane leaflet and can hydrophobically interact with the palmitoyl or myristoyl residue of proteins (32, 33). Owing to the binding energy of this hydrophobic interaction, a single palmitoyl residue can anchor more than 90% of a cytosolic protein to the plasma membrane (33). Because S-palmitoylation is reversible, a protein can shuttle between the cytosol and the plasma membrane depending on the availability of reduced sulfhydryl groups in cysteine and the adequate lipid environment in the membrane. Currently, we are working on this lipid environment to understand the mechanism by which CRPs anchor S-palmitoylated proteins and regulate protein interaction and function. In summary, the results of our study elucidate an important mechanism by which ceramide mediates interaction of cytosolic proteins with cellular membranes that is not only critical for ciliogenesis but also other contact sites between membranous compartments and microtubules.

### Data availability

All data are contained in the manuscript or the supplementary files.

## Conflict of interest

The authors declare that they have no conflicts of interest with the contents of this article.

## Supplemental data

Supplemental Movie S1 Video of *C. reinhardtii* with inhibition of S-palmitoylation by 50 μM 2BPA

Supplemental Movie S2 Video of *C. reinhardtii* without inhibition of S-palmitoylation by 50 μM 2BPA
